# Descriptive Study of the Clinical and Molecular Features of Epidermolysis Bullosa Patients in a Romanian European Reference Network-Skin Affiliated Reference Center

**DOI:** 10.7759/cureus.61160

**Published:** 2024-05-27

**Authors:** Alina Suru, Alexandru Cătălin Pâslaru, George Sorin Țiplica, Carmen Maria Sălăvăstru

**Affiliations:** 1 Pediatric Dermatology Discipline, Carol Davila University of Medicine and Pharmacy, Bucharest, ROU; 2 Pediatric Dermatology Department, Colentina Clinical Hospital, Bucharest, ROU; 3 Physiology and Neuroscience Discipline, Carol Davila University of Medicine and Pharmacy, Bucharest, ROU; 4 Genetics Department, Doctor Victor Gomoiu Children’s Hospital, Bucharest, ROU; 5 Second Dermatology Discipline, Carol Davila University of Medicine and Pharmacy, Bucharest, ROU; 6 Second Dermatology Department, Colentina Clinical Hospital, Bucharest, ROU

**Keywords:** skin fragility, dystrophic epidermolysis bullosa, genetic molecular analysis, inherited epidermolysis bullosa, inherited blistering condition

## Abstract

Background: During the last 10 years, in Romania, progress has been made for the welfare of patients suffering from epidermolysis bullosa (EB). In five university hospitals, affiliated with the National Program for the Treatment of Rare Diseases, highly trained specialists diagnose and treat patients with this rare condition. Regarding diagnosis, limitations still exist as immunofluorescence mapping and molecular genetic analysis are not accessible, and generally not reimbursed. Our objective is to present the experience in diagnosing EB patients at Colentina Clinical Hospital, highlighting genotype-phenotype correlations observed in our cohort of patients.

Methods: The records of the patients enrolled between 2012 and 2024 were analyzed considering clinical aspects, and, when available, immunofluorescence mapping, transmission electron microscopy, and genetic molecular analysis.

Results: Fifty-six patients were identified, of whom 31 cases were of dystrophic EB, three were of junctional EB, and 11 were of simplex EB. For 11 cases, the EB type could not be determined. Regarding EB simplex, two patients with *KRT5* mutations and three patients with *KRT14* mutations with various clinical expressions, from mild phenotype to severe forms, were identified. Three severe junctional EB patients were registered in our database and for one of the patients, two previously unreported mutations in the *LAMA3* gene were identified. Regarding dystrophic EB, 31 cases were identified, of which 25 were recessive dystrophic cases and six were dominant dystrophic cases. Molecular genetic testing was performed for 15 patients, and the most common variant was c.425A>G, identified in six cases.

Discussions: Two previously unreported mutations were identified, namely, *COL7A1* c.5416G>C, a heterozygous missense variant in a patient with a mild phenotype, mainly with nail involvement, and *COL7A1 *c.5960del, a variant that generates a frameshift in exon 72 resulting in a premature stop codon; this variant was identified in two siblings with a severe recessive dystrophic.

Conclusion: Important steps have been made in identifying the correct and complete diagnosis, as well as the characterization of EB patients addressing our reference center. The findings underscore the pivotal role of molecular genetic testing in confirming diagnoses and elucidating inheritance patterns, especially in cases with atypical presentations or de novo mutations.

## Introduction

Epidermolysis bullosa (EB) is a rare and debilitating genetic disorder that falls within the spectrum of fragility inherited skin diseases. This disorder is characterized by a heightened susceptibility to blister formation and skin erosion in response to minimal friction or trauma [1]. Genetic mutations affecting crucial proteins responsible for sustaining the structural integrity of the skin represent the cause of this condition that comprises a heterogeneous group of disorders, each associated with specific gene mutations. The severity of symptoms can vary widely, ranging from mild forms with localized blistering to severe subtypes that can lead to life-threatening complications [1-3].

The most recent international consensus (2020) reclassified inherited EB and other disorders with skin fragility. The four main EB types are EB simplex (EBS), junctional EB (JEB), dystrophic EB (DEB), and Kindler EB (KEB). Other disorders with skin fragility like peeling skin syndromes, erosive disorders, hyperkeratotic disorders, and connective tissue disorders with skin fragility are important for the differential diagnosis [2]. There have been described more than 30 subtypes of EB with different underlying molecular defects and variable clinical severity [4]. In certain circumstances, molecular genetic testing is required to distinguish between autosomal dominant and recessive inheritance, especially in families where the first case of EB appears de novo [5]. Patients who are born with widespread lesions, usually suffer from a severe type of EB and are sooner and better investigated and diagnosed, as opposed to patients with mild skin fragility that only display localized involvement of the acral sites with blisters and erosions affecting only the hands and feet [6].

## Materials and methods

The National Program for the Treatment of Rare Skin Diseases - Epidermolysis Bullosa was established in 2012 in Colentina Clinical Hospital, Bucharest, Romania. The Pediatric Dermatology Department, a European Reference Network-Skin affiliated referral center, is the main referral center in the country, therefore, data from 56 patients, children and adults, registered to our EB center between 2012 and 2024 were accessible. Data regarding age, gender, diagnosis (clinical, immunofluorescence mapping (IFM), transmission electron microscopy (TEM), molecular genetic testing), family history, skin and mucous membrane involvement, complications, and laboratory workouts were analyzed.

This descriptive, retrospective study included inpatients and outpatients, adults, and children (from 0 to 17 years old) with EB who attended our hospital from January 1, 2012, to April 1, 2024.

The aim of the study is to provide a comprehensive overview of the primary clinical characteristics of the study population and the diagnostic techniques employed. An additional aim was to provide an overview of the molecular genetic test outcomes and to identify potential associations between genotype and phenotype within our patient cohort.

The diagnosis was established by evaluating the clinical presentation, including the identification of fragility in the skin and mucous membranes, together with a thorough assessment of the patient's family history. Additional diagnostic methods, such as IFM, TEM, and molecular genetic testing, were employed where available.

Immunofluorescence mapping and transmission electron microscopy

For IFM and TEM, skin biopsies were performed for patients according to the current guidelines [5] after proper informed consent was signed. For selected cases of neonates with skin fragility, punch biopsy samples have been collected, fixed, and shipped to the Medical Center of Freiburg’s University where IFM was performed. TEM was performed in Bucharest, in the Ultrastructural Pathology and Bioimaging Laboratory of the National Institute of Pathology “Victor Babeş”.

Molecular genetic testing

DNA samples were collected from the peripheral blood of patients using a commercial kit after the patients/caregivers signed an informed consent. Panel sequencing of published genes involved in inherited EB and mentioned by the most recent classification consensus was performed [2].

Data were collected and analyzed using frequency and contingency tables in Microsoft Excel (Microsoft Corporation, Redmond, WA).

The study was performed in line with the principles of the Declaration of Helsinki. Approval was granted by the Ethics Committee of Colentina Clinical Hospital (Date: 01.04.2024/No. 7), and all the patients/caregivers signed an informed consent.

## Results

Study population

**Figure 1:**
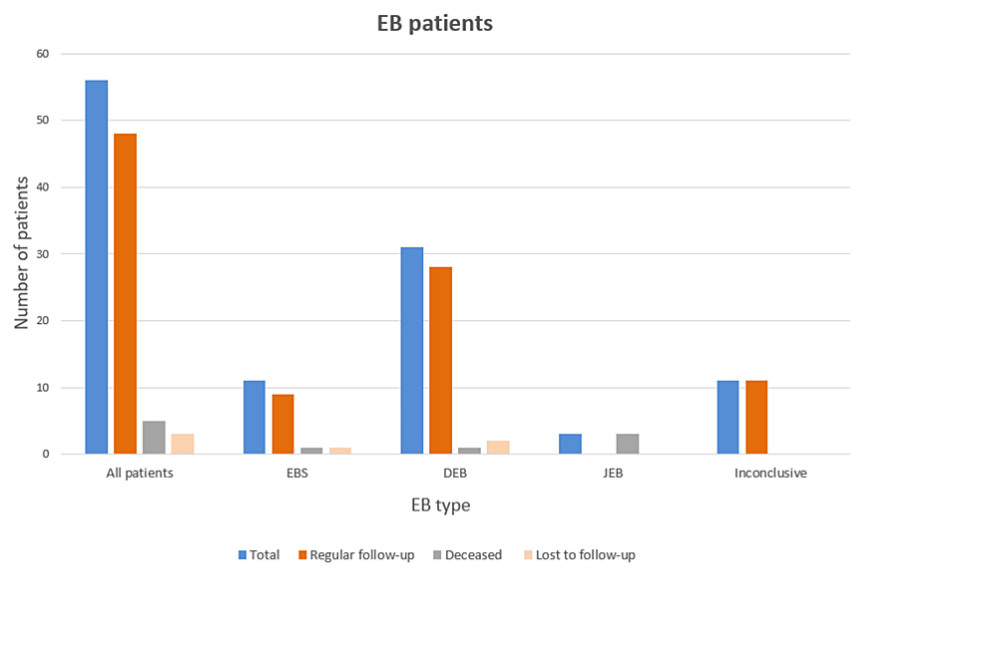
EB patients registered in Colentina Clinical Hospital in the EB program. EB: epidermolysis bullosa; EBS: epidermolysis bullosa simplex; DEB: dystrophic epidermolysis bullosa; JEB: junctional epidermolysis bullosa.

For this analysis, we considered 58 patients who were registered in the program from 2012 onwards; two patients were excluded as they were finally classified as having acquired EB and skin peeling syndrome, respectively. Of the remaining 56 cases, 31 cases were of dystrophic EB, three were of junctional EB, and 11 were of EB simplex (Table 1). For 11 cases, EB type could not be determined. Three EB patients (two DEB and one EBS) were lost to follow-up, and little data was available. The remaining 53 patients had the diagnoses described, as shown in Figure 1.

**Table 1:** Variants in epidermolysis bullosa simplex patients. AD: autosomal dominant; KRT5: gene that encodes keratin 5; KRT14: gene that encodes keratin 14; EBS: epidermolysis bullosa simplex; EBS-K, epidermolysis bullosa simplex-Koebner; EBS-WC, epidermolysis bullosa simplex Weber-Cockayne; EBS-DM, epidermolysis bullosa simplex Dowling-Meara.

Case	Sex	Inheritance	Age when tested	Gene	DNA level	Protein change	Previously reported	EBS subtype
1	F	De novo	1 week	KRT5	c.541T>C	p.(Ser181Pro)	Yes	Severe (EBS-DM)
2	F	De novo	6 months	KRT5	c.449T>C	p.(Leu150Pro)	Yes	Intermediate (EBS-K)
3	M	-	14 years	KRT14	c.373C>T	p.(Arg125Cys)	Yes	Localized (EBS-WC)
4	F	AD	2 years	KRT14	c.815T>G	p.(Met272Arg)	Yes	Intermediate (EBS-K)
5	F	-	39 years	KRT14	c.1231G>A	p.(Glu411Lys)	Yes	Severe (EBS-DM)

Regarding sex distribution, there were 30 males and 23 females, with a 1.3:1 ratio; regarding age distribution, the majority of patients were children (35), with 18 adult cases.

Twenty-nine patients had molecular genetic testing, setting a final diagnosis for 22 patients, and in seven cases, the test was inconclusive. Five patients had IFM (one EBS, two JEB, and two DEB cases), and 10 patients had TEM (three EBS, two JEB, and five DEB cases); for only two cases, one JEB patient and one intermediate recessive dystrophic EB (RDEB), all three investigations were possible.

Diagnosis

Considering the diagnosis, we registered 11 EBS cases, including three adults and eight children. From these, one patient died due to sepsis, and one was lost to follow-up. Of the nine remaining cases, five had genetic confirmation and four received a diagnosis based on the clinical aspect. The genetic variants identified for these cases are detailed in Table 1.

For three families with patients with very mild phenotypes, molecular analysis was performed. Previously unreported variants of uncertain significance (VUS) in DST, KRT14, and ITGA3 were identified (Table 2), but to this date, the cases remain unclassified.

**Table 2:** Patients with mild phenotypes where genetic testing was inconclusive. Family 1: two male siblings; family 2: father and son, family 3: mother and son. AD: autosomal dominant; DST: gene that encodes dystonin; DSP: gene that encodes desmoplakin; KRT14: gene that encodes keratin 14; ITGA3: gene that encodes integrin subunit alpha 3; VUS: variant of uncertain significance.

Family	Sex	Inheritance	Gene	DNA level	Protein change	Variant classification	Previously reported	Clinical summary
1	M	-	DST	c.10930A>G	p.(Ile3644Val)	VUS	No	Small post-vesicular erosions on hands and feet; nail dystrophy
			DSP	c.8590T>C	p.(Phe2864Leu)	VUS		
	M		DST	Alternate transcript NM_015548.4:c.9418A>G	p.(Ile3140Val)	VUS	No	The same as his sibling, also scalp hypotrichosis and keratosis pilaris
2	M	AD	KRT14	c.415G>C	p.(Ala139Pro)	VUS	No	Single bulla at birth, later mild acral skin fragility; in adulthood hand and feet atrophic scars, dyspigmentation, and nail dystrophy
	M		KRT14	c.415G>C	p.(Ala139Pro)	VUS	No	Mild acral skin fragility; post-traumatic vesicles and erosions
3	F	AD	DST	Alternate transcript NM_015548.4:c.10283A>G	p.(Glu3428Gly)	VUS	No	Very mild acral fragility, skin desquamation
			ITGA3	c.3029T>C	p.(Ile1010Thr)	VUS	No	
			ITGA3	c.394G>A	p.(Gly132Ser)	VUS	No	
	M		DST	Alternate transcript NM_015548.4:c.10283A>G	p.(Glu3428Gly)	VUS	No	The same as the mother
			ITGA3	c.3029T>C	p.(Ile1010Thr)	VUS	No	
			ITGA3	c.394G>A	p.(Gly132Ser)	VUS	No	

Three severe JEB patients were registered in our database (Table 3), including two females and one male, with skin fragility since birth and cutaneous and mucous involvement, who survived between five and 14 months, the causes of death being sepsis and failure to thrive.

**Table 3:** Variants, IFM, and TEM in junctional epidermolysis bullosa patients. IFM: immunofluorescence mapping; TEM: transmission electron microscopy; LAMA3: gene that encodes laminin subunit alpha 3; LAMB3: gene that encodes laminin subunit beta 3.

No.	Sex	Gene	Exon	DNA level	Protein level	Previously reported	IFM	TEM
1	F	LAMA3	2	c.222delG	p.(Cys75Valfs*65)	No	No	No
			24	c.3177-3178delAG	p.(Gly1061Serfs*36)	No		
2	F	LAMB3	Intronic	c.1133-22G>A	Intronic	Yes	Laminin 332 stains negative and α6β4 integrin is reduced	Yes
			8	c.727C>T	p.(Gln243*)	Yes		
3	M	-	-	-	-	-	Laminin 332 stains negative and α6β4 integrin is reduced	Yes

From our cohort of 56 patients, we identified 31 DEB cases, 18 males and 13 females, 25 RDEB, and six dominant dystrophic EB (DDEB) cases. A family with two suspected DDEB cases was lost to follow-up and one patient with RDEB died in the analyzed period. The remaining 28 cases were distributed as follows: up to one year old: two cases; one to four years old: three cases; five to nine years old: five cases; 10-14 years old: four cases; 15-19 years old: four cases; 20-24 years old: one case; 25-59 years old: eight cases; 60-99 years old: one case. From the 31 DEB cases, 15 had a diagnosis that was confirmed by molecular genetic testing. The identified variants, IFM, and TEM aspects, as well as clinical manifestations of patients, are available in Tables 4, 5.

**Table 4:** Variants, IFM, and TEM aspects in dystrophic epidermolysis bullosa patients. * Cases 5 & 6 are siblings IFM: immunofluorescence mapping; TEM: transmission electron microscopy; AR: autosomal recessive; AD: autosomal dominant; DEB: dystrophic epidermolysis bullosa; RDEB: recessive dystrophic epidermolysis bullosa; DDEB: dominant dystrophic epidermolysis bullosa.

No.	Sex	Inheritance	Exon	COL7A1 DNA level	Protein change	Previously reported	DEB subtype	IFM	TEM	
1	F	AR	3	c.425A>G	p.(Lys142Arg)	Yes	Severe RDEB	No	No	
			103	c.7723G>A	p.(Gly2575Arg)	Yes				
2	F	AR	111	c.8233C>T	p.(Arg2745*)	Yes	Severe RDEB	No	No	
			47	c.4667del	p.(Lys1556Argfs*154)	Clinvar, variation ID: 2707677				
3	M	AD	3	c.425A>G	p.(Lys142Arg)	Yes	Intermediate DDEB	No	Cleavage is present beneath lamina densa	
4	M	AD	62	c.5416G>C	p.(Gly1806Arg)	No	Localized DEB (nail only)	No	No	
5*	M	AR	4	c.497dup	p.(Val168Glyfs*12)	Yes	Severe RDEB	No	No	
			72	c.5960del	p.(Gln1987Argfs*18)	No				
6*	M	AR	4	c.497dup	p.(Val168Glyfs*12)	Yes	Severe RDEB	No	No	
			72	c.5960del	p.(Gln1987Argfs*18)	No				
7	F	AR	100	c.7547dup	p.(Asp2518*)	Clinvar, variation ID: 1448969	Intermediate RDEB	Reduced staining for collagen VII at blister roof	Cleavage beneath lamina densa; anchoring fibrils are absent on extensive areas or are very scarcely visible	
			86	c.6788G>T	p.(Gly2263Val)	Yes				
8	F	AR	73	c.6081delC	p.(Pro2029Leufs*17)	Yes	Intermediate RDEB	Reduced staining for collagen VII at blister roof	No	
			74	c.6205C>T	p.(Arg2069Cys)	Yes				
9	M	AR	3	c.425A>G	p.(Lys142Arg)	Yes	Severe RDEB	No	Absent epidermis; rare hemi desmosomes, no clear anchoring fibrils	
			4	c.497dup	p.(Val168Glyfs*12)	Yes				
10	M	AR	15	c.2005C>T	p.(Arg669*)	Yes	Intermediate RDEB	No	No	
			5	c.553C>T	p.(Arg185*)	Yes				
11	F	AR	3	c.425A>G	p.(Lys142Arg)	Yes	Severe RDEB	No	Cleavage zone beneath the lamina densa, anchoring fibrils absent. The basal membrane is interrupted in places	
12	F	AR	17	c.2305_2314delinsTT	p.(Val769Phefs*3)	Yes	Severe RDEB	No	No	
			3	c.425A>G	p.(Lys142Arg)	Yes				
13	M	AD	85	c.6727del	p.(Ser2243Leufs*145)	Clinvar, variation ID: 2118010	Intermediate DDEB	No	No	
										14	M	AR	3	c.425A>G	p.(Lys142Arg)	Yes	Severe RDEB	No	Cleavage is present beneath lamina densa. The basement membrane shows small areas of interruption. Basal keratinocytes have a reduced number of hemi desmosomes. The anchoring fibrils of the hemi desmosomes are largely absent	
4	c.497dupA	p.(Gln166GlnfsX13)	Yes																
15	F	AR	74	c.6205C>T	p.(Arg2069Cys)	Yes	RDEB-inversa	No	No	
				c.4341+2T>C	p.(?)	Clinvar, variation ID: 947463				

**Table 5:** Variants and clinical manifestations in dystrophic epidermolysis bullosa patients. * Cases 5 & 6 are siblings; ** case 11 associates minor β thalassemia. Blisters: generalized +++, widespread ++, localized +; Nails: total anonychia +++, partial anonychia ++, nail dystrophy +; anemia: mild + (10-12 g/dl), moderate ++ (8-10 mg/dl), severe +++ (< 8 mg/dl). DEB: dystrophic epidermolysis bullosa; RDEB: recessive dystrophic epidermolysis bullosa; DDEB: dominant dystrophic epidermolysis bullosa; SCC: squamous cell carcinoma.

No.	DEB subtype	*COL7A1* DNA level	Clinical manifestations								
			Blisters +/++/+++	Oral involvement microstomia/ankyloglossia		Esophageal stenosis	Joints contractures/pseudo syndactyly		Nails	Anemia	SCC
1	Severe RDEB	c.425A>G, c.7723G>A	+++	+	+	+	+	+	+++	+	-
2	Severe RDEB	c.8233C>T, c.4667del	++	+	+	+	+	+	+++	+	+
3	Intermediate DDEB	c.425A>G	+	-	-	-	-	-	++	-	-
4	Localized DEB (nail only)	c.5416G>C	+/-	-	-	-	-	-	+	-	-
5*	Severe RDEB	c.497dup, c.5960del	++	+	+	+	+	-	+++	++	-
6*	Severe RDEB	c.497dup, c.5960del	++	+	+	+	+	-	+++	+	-
7	Intermediate RDEB	c.7547dup, c.6788G>T	++	-	-	-	+	-	++	++	-
8	Intermediate RDEB	c.6081delC, c.6205C>T	++	+	+	-	+	-	+++	+	-
9	Severe RDEB	c.425A>G, c.497dup	++	+	+	+	+	-	+++	-	-
10	Intermediate RDEB	c.2005C>T, c.553C>T	++	+	+	-	+	-	+++	++	-
11	Severe RDEB	c.425A>G	+++	+	+	+	+	+	+++	+++**	-
12	Severe RDEB	c.2305_2314delinsTT c.425A>G	++	+	+	+	+	+	+++	+	-
13	Intermediate DDEB	c.6727del	++	+	+	-	+	+	+++	+	-
14	Severe RDEB	c.425A>G, c.497dupA	+++	+	+	+	+	+	+++	+	-
15	RDEB-inversa	c.6205C>T, c.4341+2T>C	+	-	+	-	-	-	+	+	-

## Discussion

Epidermolysis bullosa simplex

In our cohort of EBS patients, we identified two patients with *KRT5* variants and three patients with *KRT14* variants with various clinical expressions, from a mild phenotype with localized blisters affecting the acral areas, nail dystrophy, and dyspigmentation (case 3, Table 1), to intermediate forms (cases 2 & 4, Table 1) that displayed herpetiform blistering with erosions that were more widespread to severe forms (cases 1 and 5, Table 1), with important skin fragility with blisters and erosions on large areas of the skin.

Case 1 displayed a *KRT5* heterozygous de novo variant (c.541T>C), previously reported [7,8], resulting in a serine to proline substitution in the highly conserved helix initiation motif within the 1A domain of keratin 5 p.(Ser181Pro). Targeted parent testing was negative indicating the variant had occurred de novo; severe *KRT5*-related disease is known to be caused by a de novo variant.

Case 2 also featured a de novo heterozygous missense mutation of a single nucleotide c.449T>C, in exon 1 of *KRT5*, also reported [9], that resulted in the amino acid substitution, p.(Leu150Pro), in the head domain of keratin 5.

Case 3 presented a known heterozygous sequence change (c.373C>T) [10] that replaced arginine with cysteine at codon 125 of the *KRT14* protein (p.Arg125Cys). In the literature, this variant was shown to determine a severe generalized phenotype when it appeared de novo in a patient [11] or was autosomal dominant (AD) inherited [12]. However, our patient displayed a localized form of EBS. Familial studies are necessary to determine the path of inheritance in this case.

Case 4 also featured a heterozygous *KRT14* missense variant in c.815T>G, which determined the substitution of methionine with arginine in codon 272, which lies in the middle of the central rod domain in the linker region (L12) of the encoded keratin protein. This variant was already reported and associated with an intermediate EBS phenotype, AD inherited [13]; this was also true for our patient who came from a family with at least three other family members being affected.

Case 5 showed a well-known heterozygous variant in exon 6 of *KRT14* c.1231G>A, which substitutes amino acid glutamate (E) for lysine (K) at position p.Glu411Lys; this is consistent with severe EBS (Dowling-Meara) [14]. Family studies would allow us to establish the inheritance of the variant, as in literature was described as de novo.

Junctional epidermolysis bullosa

From the three JEB patients, one patient (case 1, Table 3) was diagnosed based on molecular genetic testing that identified two previously unreported variants in the *LAMA3* gene. The first variant was a deletion of guanine on exon 2 of *LAMA3* at position c.222del G resulting in a frameshift and a premature stop-codon c-terminal defined as p.Cys75Valfs*65 on protein level. The second deletion was a 2bp-deletion of adenine and guanine on exon 24 of *LAMA3* at position c.3177-3178del AG resulting in a frameshift and a pre-terminal stop-codon, defined as p.(Gly1061Serfs*36) on protein level. The first variant was inherited from the father and the second from the mother.

The second patient (case 2, Table 3) had a complete diagnosis with IFM and TEM suggestive of JEB, and molecular testing showed two variants that have previously been observed in patients with JEB. The first was an intron splice-site mutation (*LAMB3*, c.1133-22G>A) cited in patients with origins in the Balkan area [15]; the second was a variant in exon 8, c.727C>T, that creates a premature stop codon p.(Gln243*) in the *LAMB3* gene that is expected to result in an absent or disrupted protein product [16,17].

The third patient (case 3, Table 3) was diagnosed based on the clinical features, IFM (laminin 332 stained negative) and TEM (cleavage in lamina lucida).

Dystrophic epidermolysis bullosa

DEB cases were the most frequent cases, as mild cases of EBS were potentially less likely to be referred to specialized centers, which could account for this result.

Regarding the 15 DEB patients that were analyzed, the most common variant was *COL7A1* c.425A>G identified in six cases (cases 1, 3, 9, 11, 12, and 14, Tables 4, 5); c.425A>G is a well-known hotspot mutation [18-21] located in exon 3, changing nucleotide adenine to guanine and resulting in an amino acid change of lysine to arginine at protein level defined as p.(Lys142Arg); as a consequence, aberrant splicing occurs. The inheritance pattern for this variant is consistent with autosomal recessive inheritance. From the six cases, five were associated with severe RDEB and one with intermediate DDEB.

The first patient (case 1, Tables 4, 5) had two well-known *COL7A1* variants, c.425A>G, mentioned above, and c.7723G>A, a change of the nucleotide guanine to adenine in exon 103 leading to a change of the amino acid glycine to arginine at position p.(Gly2575Arg) on protein level [20]. This patient associated a severe phenotype with generalized blisters, large erosions affecting the posterior trunk, microstomia, ankyloglossia, esophageal stenosis since a young age, failure to thrive, joint contractures, pseudo syndactyly, and anemia.

Another known variant identified in our cohort was *COL7A1* c.497dup [22-24]. This variant generates a frameshift in exon 4 resulting in a premature stop codon and was reported in patients with RDEB. This was identified in three patients (cases 5, 6, and 9, Tables 4, 5), all with severe RDEB phenotypes. Case 14 had a similar heterozygous variant, c.497dupA [25], which resulted in a frameshift that determined a premature stop codon defined as p.Gln166Glnfs*13.

A heterozygous missense variant *COL7A1* c.6205C>T was identified in two patients. This variant can be associated with intermediate RDEB or RDEB inversa [18,26]. One patient (patient 8, Tables 4, 5) associated another known variant, c.6081delC [27], which determined a clinical picture suggestive of intermediate RDEB with blisters and erosions, mainly in the posterior trunk, axillae, buttocks, alopecia, esophageal involvement, and nail involvement, but without mitten formation. Another, *COL7A1* compound heterozygous patient (case 15, Tables 4, 5), associated with a previously poorly characterized variant, c.4341+2T>C, and a clinical picture suggestive of RDEB inversa with mild blistering at birth, nail dystrophy in childhood, and later blisters that affected the buttocks, genital area, mild esophageal involvement, ankyloglossia, and nail dystrophy.

Another patient (case 2, Tables 4, 5) presented two heterozygous variants, a nonsense variant *COL7A1* c.8233C>T, p.(Arg2745*) and a frameshift, splice region variant *COL7A1* c.4667del, p.(Lys1556Argfs*154). The first variant changes arginine with a premature stop codon in exon 111 and was previously reported in patients with RDEB [18,28,29]. The second less known variant generates a frameshift in exon 47 resulting in a premature stop codon. This patient associated severe RDEB features with widespread blisters and erosions, microstomia, ankyloglossia, esophageal stenosis, pseudo syndactyly, total anonychia, anemia, and squamous cell carcinoma in hand mitten deformities at the age of 19.

Another patient (case 4, Tables 4, 5) had a heterozygous missense variant *COL7A1* c.5416G>C, p.(Gly1806Arg), previously unreported, and associated a mild phenotype with nail dystrophy of the toes, as well as of two fingers that started when the patient was nine months old, and finger blisters that appeared later at nine years old.

Two *COL7A1* compound heterozygous patients who were siblings (cases 5 and 6, Tables 4, 5) associated c.497dup [22,23], and another previously unreported variant, c.5960del. This variant generates a frameshift in exon 72 resulting in a premature stop codon. The siblings featured skin fragility since birth, disseminated blisters, mucosal involvement, and failure to thrive.

Patient 7 had two heterozygous *COL7A1* variants c.7547dup and c.6788G>T. The first less-known sequence change creates a premature translational stop signal p.(Asp2518*). It is expected to result in an absent or disrupted protein product. Loss-of-function variants in *COL7A1* are known to be pathogenic [24]. The second reported variant [21], replaces glycine with valine at codon 2263 of the *COL7A1* gene p.(Gly2263Val). The patient displayed blisters and erosions on the neck, lumbar area, and limbs (elbows, hands, knees, calves, and feet), anemia, and finger contractures.

For patient 10 (Tables 4, 5), two previously described pathogenic variants, c.2005C>T p.(Arg669*) [30] and c.553C>T p.(Arg185*) [20], were identified in *COL7A1*. The clinical picture showed erosions on the trunk and limbs, microstomia, ankyloglossia, digit fusions, and anemia.

Patient 12 presented the well-characterized variant *COL7A1* c.425A>G, detailed above, but also c.2305_2314delinsTT. This known variant [18] creates a premature translational stop signal p.(Val769Phefs*3), which is expected to result in an absent or disrupted protein product. This patient associated blisters and erosions on the neck, elbows, knees, hands, and legs, microstomia, ankyloglossia, esophageal stenosis, joint contractures, pseudo syndactyly, and total anonychia.

Patient 13 (Tables 4, 5) had a poorly described *COL7A1* heterozygous frameshift variant, c.6727del, that creates a premature translational stop signal p.(Ser2243Leufs*145). The clinical picture is represented by blisters and erosions on the elbows, knees, hands, and legs, joint contractures, pseudo syndactyly, and total anonychia.

Limitations of the study

Although we received cases from different regions of the country, the research does not include the entire EB population in Romania. Furthermore, it is important to note that the study was conducted in an observational manner. Regarding the risk of misclassification, patients could have been misclassified regardless of the center's important expertise. Among the cohort of patients designated as "inconclusive" (n = 11, Figure 1 and Table 2), a definitive diagnosis was not possible even with DNA analysis. This could potentially be attributed to technical deficiencies, the existence of atypical variants, variants in genes unknown to be associated with EB at the time of diagnostics, or variants in genes that have yet to be identified. There have been advancements in the classification and (sub)classification of the various subtypes of EB over time. This may be especially true regarding EBS.

## Conclusions

The present study explores the clinical, molecular, and genetic aspects of EB, shedding light on its diverse manifestations and diagnostic challenges. Through analysis of patient data spanning over a decade, the study furthers the understanding of the heterogeneity of EB subtypes, ranging from mild localized forms to severe, life-threatening conditions.

Important steps have been taken in identifying the correct and complete diagnosis and characterization of the EB patients addressing our reference center. These efforts have led to the identification of two new genetic variants in three DEB cases, two new variants in a JEB patient, and a better correlation between four poorly characterized variants in DEB and phenotype. The findings underscore the pivotal role of molecular genetic testing in confirming diagnoses and elucidating inheritance patterns, especially in cases with atypical presentations or de novo variants.
